# Relaxed vigilance: humor as a regulatory capacity in care systems

**DOI:** 10.3389/fsoc.2026.1795254

**Published:** 2026-07-27

**Authors:** Lawrence Merle Nelson

**Affiliations:** Mary Elizabeth Conover Foundation, Inc., McLean, VA, United States

**Keywords:** healthcare teamwork, humor in healthcare, laughter and social signaling, psychological safety, relaxed vigilance, safe harboring, signal detection in clinical care, team communication

## Abstract

Sustaining vigilance over time presents a regulatory challenge for care systems operating under conditions of uncertainty. When vigilance becomes chronically elevated, it may narrow attention, erode relational trust, and reduce adaptive capacity. This Perspective introduces relaxed vigilance, a regulatory condition in which vigilance remains active while cognitive flexibility and relational coordination are preserved. The article examines humor, particularly laughter, as a biologically and socially grounded process that may signal or support this regulatory balance. Drawing on literature from evolutionary biology, stress physiology, medical sociology, patient safety culture, and healthcare teamwork, the framework distinguishes relaxed vigilance from adjacent constructs, including psychological safety, situational awareness, resilience, and burnout. Within clinical and organizational contexts, laughter may function as an interactional signal indicating that attention to risk persists while relational conditions necessary for collaboration remain intact. The framework emphasizes that this relationship is conceptual rather than causally established, and that organizational conditions such as hierarchy, workload, reporting culture, and communication structures shape whether relaxed vigilance can emerge or be sustained. In caregiving systems, where clinicians operate under a continuing duty of care, sustaining this balance may help preserve attention to risk while supporting trust, communication, and adaptive coordination under prolonged uncertainty.

## Introduction

Moments of acute vigilance often arise in response to immediate threat and highlight a fundamental challenge for care systems: how to sustain attention to risk without tipping into fear, rigidity, or fragmentation. While vigilance is essential for recognizing emerging threats, its persistence over time can introduce regulatory pressures that reshape social interaction, institutional coordination, and individual judgment. Systems responsible for care and public safety must remain attentive under prolonged uncertainty, yet excessive or unmodulated vigilance may narrow communication, reduce conversational openness, and undermine adaptive coordination within teams. In clinical and organizational environments, communication breakdown and defensive interaction patterns have been shown to impair teamwork and situational awareness, both of which are essential to maintaining safe, coordinated care (Lin et al., 2022; Arad et al., 2022). The challenge, therefore, lies not simply in intensifying vigilance, but in understanding how vigilance itself can remain regulated so that attention to risk is sustained without eroding the relational and communicative conditions required for coordinated action.

Across biological and social systems, vigilance mechanisms evolved to detect threats and mobilize coordinated responses. These mechanisms, however, are not designed for continuous activation. When vigilance becomes chronically elevated, sustained physiological arousal may impair cognitive flexibility, social attunement, and cooperative problem-solving. Research in evolutionary biology and neuroscience suggests that affiliative vocalizations, such as laughter, are associated with modulation of stress-related physiological processes and the maintenance of social bonds within groups (Dunbar et al., 2012; Davila-Ross et al., 2011). Studies examining humor and laughter in health contexts likewise suggest that these responses are associated with reduced anxiety and improved emotional regulation under certain conditions (Zhao et al., 2019). Rather than signaling disengagement from risk, laughter often appears in moments of uncertainty, hierarchy strain, or tension within groups—contexts in which vigilance remains necessary but excessive escalation would be costly. These observations suggest that laughter may function as a *regulatory signal*, allowing attention to remain active while releasing accumulated tension and restoring relational flexibility. This Perspective describes the resulting regulatory condition as *relaxed vigilance.*

The concept of relaxed vigilance provides a way to interpret how vigilance may remain active without becoming destabilizing. In clinical teams and other cooperative care settings, maintaining this balance requires the capacity to sustain attention to potential risk while preserving the relational conditions necessary for communication, coordination, and trust. Laughter and humor may play a role in this process by signaling affiliative intent and reinforcing social cohesion within groups (Dunbar et al., 2021). At the same time, humor may have varied effects depending on context, timing, audience, and power relations within groups (Raecke and Proyer, 2024; Fang et al., 2024; Rosenberg et al., 2024). Taken together, these findings suggest that humor and laughter should not be understood merely as personality traits or informal coping strategies, but as *observable social signals* that may indicate whether regulatory processes within a team remain intact. This Perspective, therefore, examines laughter as a potential indicator of relaxed vigilance across biological, interpersonal, and institutional levels. While prior research has examined humor, laughter, and psychological safety in healthcare teams, the present framework proposes relaxed vigilance as a regulatory state linking biological stress modulation, interactional signaling, and institutional coordination in care systems.

## Conceptual framework: relaxed vigilance

Relaxed vigilance is a regulated state in which attention to potential threat is sustained without rigidity, exhaustion, or loss of relational coherence. Unlike hypervigilance, which reflects persistent defensive arousal and narrowing of attention, relaxed vigilance allows systems to remain perceptive while preserving cognitive flexibility, social connection, and coordinated response. This balance enables individuals and groups to remain attentive to emerging risk while maintaining the relational conditions necessary for communication, judgment, and collective action.

Across biological and social systems, vigilance mechanisms evolved to detect threats and mobilize responses, yet they typically function episodically rather than continuously. When activation becomes chronic, sustained physiological arousal may impair learning, judgment, and relational attunement. Research on laughter and affiliative vocalizations suggests that these signals may help regulate arousal while maintaining social bonding within groups (Dunbar et al., 2012; Davila-Ross et al., 2011). In human interaction, such signals often appear in situations involving elevated arousal that nonetheless remain non-threatening, such as play, social negotiation, or moments of tension within cooperative groups. These patterns suggest that laughter may help restore flexibility within vigilance by allowing physiological activation to decrease while attention to the surrounding context remains intact.

In this sense, relaxed vigilance is not merely a psychological state but a regulatory condition of functioning care systems, allowing vigilance to remain active without destabilizing the social and communicative processes on which coordinated care depends. The concept, therefore, provides an analytical lens for examining how biological regulation, interactional dynamics, and institutional practices interact to sustain vigilance over time. Rather than diminishing awareness, relaxed vigilance may allow weak signals of concern to be perceived and interpreted with greater flexibility during clinical interactions.

## Humor and laughter: conceptual distinction

Humor and laughter are related but distinct phenomena. Humor refers to a cognitive or social stimulus involving incongruity, tension, or reinterpretation of a situation, whereas laughter is a physiological and social response that may follow such stimuli. Humor can occur without laughter, and laughter may occur in the absence of humor, particularly in affiliative or tension-regulating interactions. Because laughter is observable and socially shared, it provides a useful signal for examining regulatory processes within groups.

Research examining laughter in social and biological contexts suggests that it functions as a form of affiliative signaling that supports social bonding and group cohesion (Dunbar et al., 2012; Dunbar et al., 2021). In healthcare environments, different humor styles have been shown to relate differently to workplace functioning and stress, indicating that humor can serve both supportive and harmful roles depending on context (Raecke and Proyer, 2024; Fang et al., 2024). Organizational research similarly suggests that the effects of humor depend strongly on timing, audience awareness, and power relations within groups (Rosenberg et al., 2024). For these reasons, the present framework focuses primarily on laughter as an observable signal that regulatory processes within a group remain active, while recognizing that humor itself may take multiple forms and produce varied outcomes.

## Regulatory balance in vigilance

**Figure 1:**
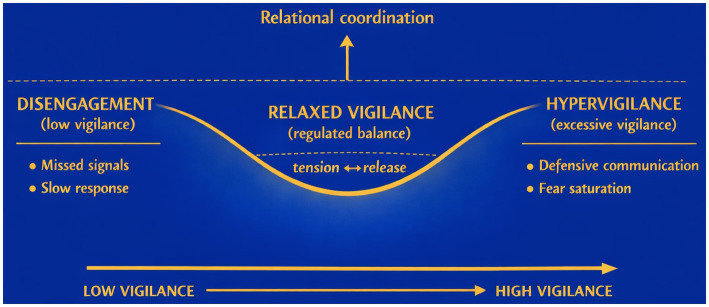
Relaxed vigilance as a regulatory spectrum in care systems. Vigilance in care systems operates along a continuum between two regulatory failure states: insufficient vigilance (disengagement) and excessive vigilance (hypervigilance). Disengagement is characterized by reduced situational awareness, missed signals, and fragmented coordination, whereas hypervigilance is associated with defensive communication, rigidity, and fear saturation. Relaxed vigilance occupies the regulated middle range, in which attention to potential threats remains active while communication, relational trust, and coordinated responses are preserved. Within this regulated zone, affiliative signals such as laughter may help release accumulated tension without disengaging attention, supporting the restoration of conversational openness and relational safety within teams.

**Figure 2:**
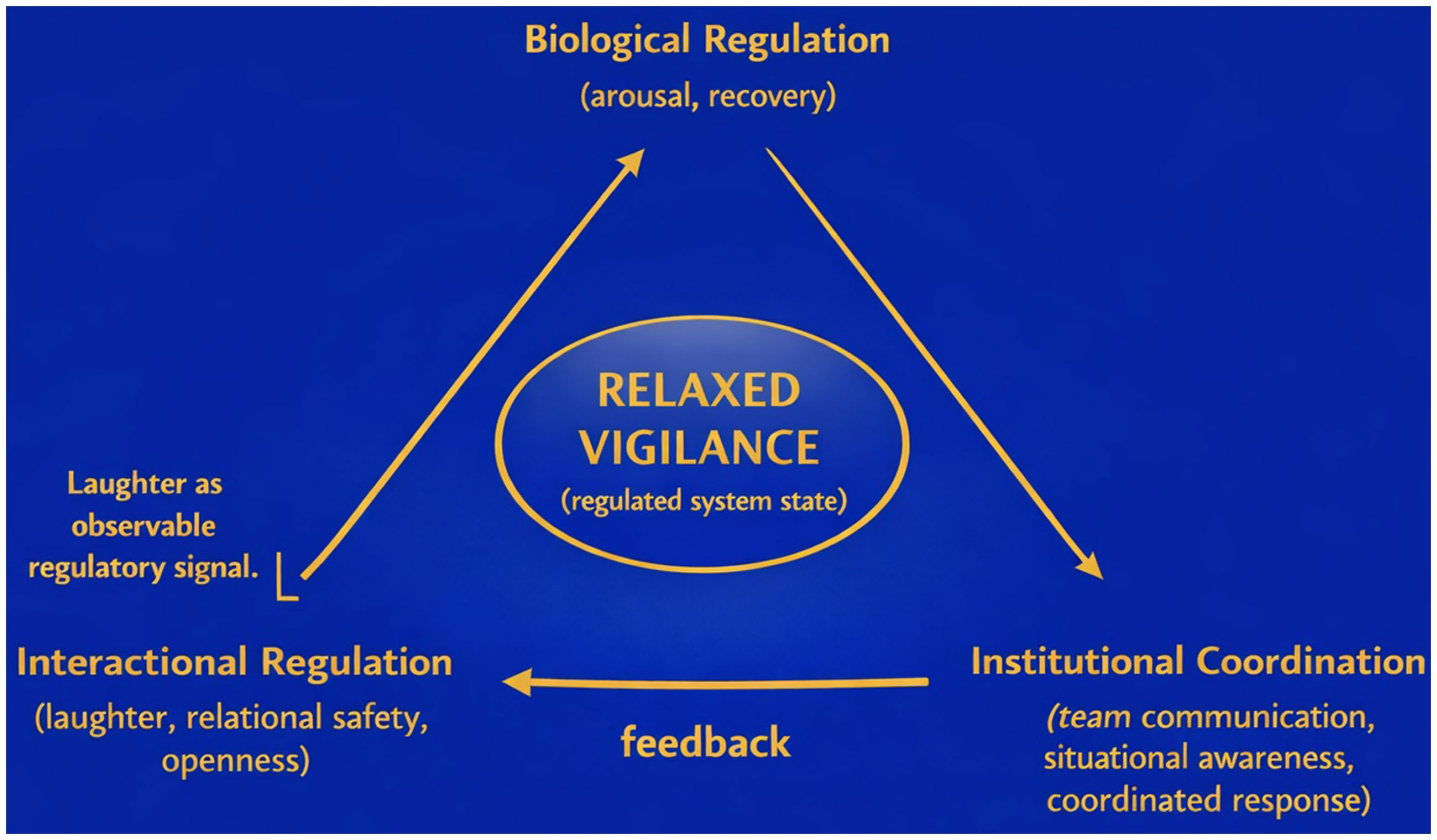
Multilevel regulation of relaxed vigilance in care systems. Relaxed vigilance emerges from interacting regulatory processes operating across biological, interactional, and institutional levels. At the biological level, systems oscillate between arousal and recovery as part of normal physiological regulation. At the interactional level, signals such as laughter, relational safety, and conversational openness help regulate tension within group communication. At the institutional level, coordination mechanisms such as team communication, situational awareness, and structured collaboration support sustained vigilance in complex care environments. The figure emphasizes the interactional and institutional pathways through which regulatory processes may influence one another. The model is intended as a conceptual representation rather than a comprehensive mapping of all possible feedback relationships among biological, interactional, and institutional levels.

Within this framework, relaxed vigilance can be understood as a regulatory balance between excessive and insufficient vigilance. At one extreme, persistent threat activation may produce hypervigilance, characterized by rigidity, defensive narrowing of attention, and exhaustion. At the opposite extreme, insufficient vigilance may lead to disengagement, loss of situational awareness, or erosion of coordinated response. Relaxed vigilance occupies the regulated middle range, where attention to potential threat remains active while cognitive flexibility, communication, and relational trust are preserved. In human groups, particularly in caregiving systems that must sustain attention under ongoing strain, regulatory signals that allow tension to be released without disengagement may help maintain this balance. Laughter has been associated with physiological recovery following heightened arousal and with maintaining social bonding within groups (Dunbar et al., 2012; Dunbar et al., 2021). For this reason, laughter may function as one observable signal that vigilance remains regulated rather than rigid or depleted (Figure 1). Together, Figures 1, 2 illustrate both the regulatory spectrum within which vigilance operates and the multilevel processes through which care systems maintain vigilance without collapsing into disengagement or hypervigilance.

## Illustrating relaxed vigilance in clinical interaction

To illustrate how relaxed vigilance may appear within everyday clinical interaction, the following composite scenario depicts a moment in which vigilance remains active while relational tension is regulated within a healthcare team. The following vignette is presented as an illustrative example rather than as empirical case data.

During a multidisciplinary clinical huddle late in the afternoon, the discussion grows increasingly tense as the team reviews a near-miss from the previous night. Voices tighten, posture stiffens, and the room grows quiet as participants review the sequence of events that nearly resulted in patient harm. After a long pause, one clinician looks around the room and remarks, “At least we’re still talking to each other.” The comment prompts brief laughter among several team members, followed by a collective exhale, and the discussion resumes with greater openness and focus.

The laughter does not resolve the error or diminish its seriousness. Within the logic of this illustrative example, it may be interpreted as releasing accumulated tension sufficiently for the group to continue examining the event together. Such a moment could permit conversation to become less guarded and encourage renewed participation in discussion. In this interpretation, vigilance remains fully engaged while the interaction shifts from defensive silence toward collaborative inquiry. The vignette is intended to illustrate a possible regulatory process rather than to demonstrate a specific causal effect.

Research on communication within healthcare teams suggests that relational trust and psychological safety influence clinicians’ ability to speak openly about risks, near misses, and uncertainty (O’Donovan et al., 2021; Dietl et al., 2023). Similarly, studies of team coordination indicate that brief, structured interactions, such as clinical huddles, support situational awareness and collective problem-solving within complex care environments (Lin et al., 2022). In this context, laughter may function as a micro-regulatory signal that tension has not escalated into interpersonal threat, allowing attention to remain directed toward the clinical problem rather than toward defensive self-protection. Because the vignette is illustrative rather than empirical, these interactional outcomes should be understood as theoretically plausible interpretations consistent with the proposed framework rather than as demonstrated findings.

## Social mechanisms of regulatory laughter

From a sociological perspective, the significance of laughter in such moments lies less in amusement than in its interactional effects. Laughter can mark a temporary shift in the emotional tone of an interaction, signaling that tension has not escalated into interpersonal threat. When this occurs, conversational dynamics often change in observable ways: turn-taking becomes more fluid, participants appear less defensive, and individuals may re-engage in discussion after hesitations or silences.

Studies of communication within healthcare teams indicate that relational trust and psychological safety influence clinicians’ ability to speak openly about uncertainty, risk, and potential error (O’Donovan et al., 2021; Dietl et al., 2023). When relational safety is perceived as fragile, participants may withhold information, avoid disagreement, or limit their contributions to protect themselves or others. Under such conditions, vigilance may persist at the individual level while collective learning and coordination become constrained.

Within this interactional context, brief shared laughter may contribute to a shift in conversational framing. Rather than signaling dismissal of the problem, laughter can mark a moment in which participants recognize their continued alignment as a group despite tension or uncertainty. This shift may allow discussion to move from a defensive or blame-oriented frame to one focused on collective problem-solving. In this sense, laughter can function as a micro-level regulatory signal that relational safety has been restored sufficiently for collaborative inquiry to continue.

To situate this concept within existing scholarship on healthcare teamwork and safety culture, the following section clarifies how relaxed vigilance relates to adjacent constructs such as psychological safety, team communication, and signal detection.

These interactional dynamics are also consistent with several traditions in medical and organizational sociology. Interaction rituals refer to recurring social encounters through which participants establish shared attention, emotional alignment, and a sense of collective participation (Clarke and Waring, 2018). Emotional labor refers to the effort required to manage and express emotions in ways that align with professional expectations and organizational norms (Hochschild, 1979; Wharton, 2009). Research on interaction rituals within therapeutic communities suggests that shared social practices can transform interpersonal relationships by reinforcing a sense of belonging, trust, and collective participation (Clarke and Waring, 2018). Similarly, sociological scholarship on emotional labor emphasizes that professionals continuously regulate emotional expression within institutional settings, shaping both individual well-being and group functioning (Hochschild, 1979; Wharton, 2009). Studies examining shared enjoyment and group participation further suggest that collective experiences can foster cohesion by creating shared narratives and fostering collaborative engagement (Fine and Corte, 2017). From this perspective, laughter may be understood not merely as an individual emotional response but as an interactional signal embedded within broader social processes that help regulate relationships, emotional expression, participation, and coordination in care environments.

## Conceptual boundaries of relaxed vigilance

The concept of relaxed vigilance should be distinguished from several adjacent constructs commonly discussed in healthcare teamwork and organizational research, including psychological safety, team communication, resilience, and burnout. Psychological safety describes a shared perception among team members that they can speak up, ask questions, and raise concerns without fear of interpersonal risk or punishment (Grailey et al., 2021; O’Donovan et al., 2021). Research on healthcare teams shows that psychologically safe environments are associated with greater openness in communication and improved learning behaviors (Grailey et al., 2021; Dietl et al., 2023). Closely related work on speaking up for patient safety highlights that hierarchical barriers and interpersonal risk can inhibit clinicians from raising concerns even when they recognize potential safety threats (Okuyama et al., 2014).

Relaxed vigilance differs from these constructs by describing a regulatory state within social and attentional systems rather than a property of organizational climate. In this state, individuals remain attentive to environmental and interpersonal signals while defensive hypervigilance is reduced. Rather than diminishing awareness, relaxed vigilance may allow signals to be perceived and interpreted with greater flexibility during clinical interactions. Relaxed vigilance should also be distinguished from resilience and burnout. Resilience generally refers to the capacity to adapt and recover following stress or adversity, whereas relaxed vigilance describes a regulatory condition operating during ongoing periods of uncertainty and responsibility. Burnout, by contrast, is characterized by exhaustion, depersonalization, and reduced professional efficacy. Within the present framework, burnout may be understood as one possible consequence of prolonged dysregulated vigilance, whereas relaxed vigilance represents a state in which attentiveness is sustained without progressing toward exhaustion or relational disengagement (Bartzik et al., 2021; Fang et al., 2024). Humor and shared laughter are one possible mechanism through which such regulatory states may emerge in group contexts. Research on humor and laughter suggests that these behaviors can influence social bonding, stress regulation, and perceptions of interpersonal connection within groups (Dunbar et al., 2012; Dunbar et al., 2021). Studies of humor in healthcare settings similarly suggest that humor interventions and humor styles may influence stress, work engagement, and team dynamics among health professionals (Bartzik et al., 2021; Fang et al., 2024; Raecke and Proyer, 2024).

Healthcare delivery occurs in complex environments where early signals of risk are often subtle, ambiguous, or distributed across multiple team members. Research on teamwork in healthcare emphasizes that effective communication and coordination are essential for maintaining situational awareness and detecting emerging problems within dynamic clinical settings (Manser, 2009). Similarly, studies of safety culture highlight that communication climates characterized by trust and openness facilitate the recognition and escalation of safety concerns within healthcare organizations (Churruca et al., 2021). Empirical research in clinical environments further demonstrates that psychological safety and interdisciplinary communication interventions can improve the likelihood that clinicians share information and raise concerns about potential risks (Dietl et al., 2023; O’Donovan et al., 2021).

Research in medical and organizational sociology suggests that vigilance is shaped not only by individual perception but also by the communication cultures within which clinicians work. Patient safety culture encompasses shared norms, expectations, and interactional practices that influence whether concerns are voiced, uncertainty is acknowledged, and emerging risks are collectively interpreted (Churruca et al., 2021). From this perspective, relaxed vigilance may be understood as a regulatory condition that supports participation in these communicative processes without requiring either defensive hypervigilance or disengagement. The concept, therefore, complements existing scholarship on safety culture by focusing on how vigilance itself remains socially regulated within ongoing patterns of interaction.

Within this broader literature, relaxed vigilance may describe a social–cognitive state in which attentiveness to signals is preserved while defensive interpersonal threat responses are reduced. Such a state may help support communication environments in which weak signals of concern can surface and be interpreted collaboratively by teams. Communication structures such as multidisciplinary team huddles have similarly been shown to improve coordination and shared awareness in healthcare settings (Lin et al., 2022). Together, these findings suggest that interactional dynamics—including humor, shared laughter, and other forms of social regulation—may influence how attentively clinicians monitor signals and communicate uncertainty within complex care environments.

Situational awareness refers to the perception, interpretation, and anticipation of clinically relevant information within dynamic environments (Manser, 2009). While relaxed vigilance may support situational awareness, it describes a regulatory condition rather than an information-processing capability. As a conceptual framework, relaxed vigilance is not presented as a validated measurement construct. However, observable indicators potentially consistent with relaxed vigilance may include sustained attention to risk-related information, willingness to communicate uncertainty, collaborative problem-solving following periods of tension, and affiliative signals such as shared laughter that do not disrupt task engagement. Future empirical work will be needed to determine whether relaxed vigilance can be operationalized as a distinct regulatory construct and how it relates to communication patterns, psychological safety, burnout, and team performance.

## Evolutionary and physiological foundations

Understanding laughter as a regulatory process raises a basic question: why would such a behavior persist across human societies and appear in other social mammals? From an evolutionary perspective, behaviors that are energetically costly, socially conspicuous, and reliably expressed during heightened arousal are unlikely to persist unless they confer adaptive advantages. Comparative research suggests that laughter-like vocalizations occur in other primates during play and affiliative interaction, particularly in situations involving elevated arousal that remain non-threatening (Davila-Ross et al., 2011). These observations suggest that laughter may function as a regulatory signal that releases tension while maintaining attention and restoring relational flexibility.

In humans, laughter appears to retain and elaborate this regulatory timing. Studies of social laughter indicate that it is associated with increased pain thresholds and endorphin activation, findings interpreted as reflecting mechanisms involved in social bonding and physiological recovery following periods of tension (Dunbar et al., 2012). Such findings suggest that laughter may contribute to the restoration of physiological flexibility after heightened arousal rather than simply marking amusement.

Evidence from clinical and psychological research similarly indicates that humor and laughter may be associated with changes in stress-related outcomes. A meta-analysis of humor-based interventions reported associations with reduced anxiety and depressive symptoms, and improved sleep quality among adults (Zhao et al., 2019). Although such studies involve structured interventions rather than spontaneous laughter in clinical teams, they suggest plausible pathways through which laughter may contribute to stress regulation.

Taken together, evolutionary, physiological, and behavioral research suggests that laughter may function as a regulatory signal that allows for the coexistence of tension and social engagement. Rather than terminating vigilance, laughter may support oscillation between activation and recovery, allowing individuals and groups to remain attentive while avoiding prolonged defensive arousal. Within this perspective, laughter may serve as a signal that vigilance remains regulated rather than rigid, providing one pathway through which human systems sustain adaptive responsiveness over time (Figure 2). The evidence supporting this framework comes from diverse sources, including comparative research, laboratory studies, humor interventions, and healthcare teamwork literature (Davila-Ross et al., 2011; Zhao et al., 2019; Lin et al., 2022). These sources support biological plausibility and conceptual integration, but they do not directly establish that spontaneous laughter among clinical teams leads to relaxed vigilance. The clinical application proposed here should therefore be understood as a theoretical inference requiring future empirical testing.

## Regulation and coherence in care systems

Care systems operate under conditions that require sustained vigilance over extended periods. Healthcare organizations must maintain attention to uncertainty, risk, and potential harm while coordinating complex interactions among clinicians, patients, and institutions. Under such conditions, vigilance is often operationalized through procedures, protocols, and monitoring systems designed to reduce error and improve safety. While these structures are necessary, research in healthcare organizations suggests that system performance also depends heavily on relational dynamics within teams, including communication climate, trust, and psychological safety (Arad et al., 2022; O’Donovan et al., 2021).

Studies of patient safety and teamwork indicate that environments characterized by relational safety and open communication are associated with stronger coordination and more effective detection of emerging problems (Arad et al., 2022; Dietl et al., 2023). Conversely, environments marked by defensive communication or silence may preserve outward vigilance while limiting the flow of information necessary for collective learning and adaptation. These findings suggest that institutional vigilance cannot be sustained solely through formal monitoring structures. Instead, regulatory processes operating within everyday interaction help determine whether vigilance remains adaptive or becomes rigid, fragmented, or suppressed. Organizational conditions likely influence whether relaxed vigilance can emerge or be sustained within care systems. Factors such as staffing levels, workload intensity, reporting cultures, professional hierarchies, and communication structures shape how clinicians experience and respond to uncertainty. Environments characterized by excessive workload, punitive responses to error, or restricted opportunities for communication may encourage defensive forms of vigilance while reducing relational flexibility and collaborative problem-solving. Conversely, institutions that support psychological safety, structured communication, interdisciplinary collaboration, and learning-oriented responses to uncertainty may create conditions that help sustain vigilance without escalating fear or disengagement. From this perspective, relaxed vigilance is not solely an individual or team-level phenomenon but may also reflect the regulatory capacities of organizations themselves.

Within this context, humor and laughter may occasionally function as indicators of regulatory capacity within teams and organizations. Research examining humor in healthcare workplaces suggests that humor can influence stress perception, work engagement, and the experience of meaning in high-responsibility work environments (Bartzik et al., 2021). At the same time, sociological and organizational research indicates that humor is not uniformly beneficial. Different humor styles may support affiliation and resilience or reinforce hierarchy and exclusion depending on context and power relations (Fang et al., 2024; Rosenberg et al., 2024). For this reason, the presence or absence of humor must be interpreted cautiously, as a contextual signal rather than as a universal marker of system health.

Within care systems, the need for ongoing vigilance is closely linked to the professional responsibility to attend to others’ well-being. Clinical work places individuals and teams in positions where decisions carry meaningful consequences for patient safety, recovery, and trust. Under these conditions, the maintenance of attentive, coordinated response is not only a technical requirement but also a structural feature of caregiving institutions. The capacity of clinicians and teams to remain perceptive to emerging risks while continuing to communicate, learn, and coordinate can therefore be understood as an institutional expression of *duty of care*. From this perspective, sustaining relaxed vigilance may represent one way care systems maintain their duty of care over time—remaining attentive to emerging risks while preserving the relational conditions necessary for communication, judgment, and coordinated response. Regulatory processes that preserve relational trust, psychological safety, and communication openness help sustain this capacity over time. When such processes remain active, vigilance may continue without collapsing into fear, rigidity, or withdrawal, allowing care systems to maintain coherence while confronting uncertainty and strain.

## When humor fails or harms

Humor and laughter do not uniformly function as regulatory signals of safety or cohesion. Sociological research shows that humor can also reinforce hierarchy, signal exclusion, or operate as ridicule depending on context and power relations (Rosenberg et al., 2024; Fang et al., 2024). In professional environments such as healthcare, humor may therefore support affiliation in some circumstances while contributing to stigma, boundary maintenance, or interpersonal discomfort in others. For this reason, the absence of humor should not automatically be interpreted as a failure of regulatory capacity. Moments of silence or seriousness may instead reflect cultural norms, professional boundaries, or the gravity of particular clinical situations. Interpreting humor as social data, therefore, requires attention to timing, audience, and institutional context.

This concern is especially important in hierarchical relationships. Healthcare organizations are structured by differences in authority, professional status, expertise, and accountability, all of which shape how communication is interpreted and enacted. Under these conditions, humor can facilitate connection and mutual understanding, but it can also reinforce existing power arrangements. Individuals in positions of authority may use humor to define acceptable boundaries of participation, signal approval or disapproval, or redirect attention away from uncomfortable topics. Conversely, individuals in subordinate positions may feel pressure to laugh, remain silent, or avoid challenging prevailing interpretations in order to preserve social standing or avoid interpersonal risk. The meaning of laughter, therefore, cannot be separated from the power relationships within which it occurs.

From this perspective, laughter should not be interpreted as a universal indicator of psychological safety, organizational health, or effective communication. A group may laugh while avoiding difficult conversations, masking disagreement, or reinforcing social boundaries. Conversely, serious or emotionally restrained interactions may still reflect high levels of trust, respect, and collaborative functioning. The present framework, therefore, treats humor and laughter as context-dependent social signals whose significance emerges through interaction rather than as inherently positive phenomena. Whether humor contributes to relaxed vigilance or undermines it depends on factors including hierarchy, audience, timing, intent, and organizational culture (Clarke and Waring, 2018; Rosenberg et al., 2024).

An additional limitation concerns the directionality of the relationship between laughter and relaxed vigilance. The present framework proposes that laughter may function as a regulatory signal that maintains or restores relational flexibility in the face of uncertainty. However, the available evidence does not establish whether laughter contributes to the emergence of relaxed vigilance or whether groups already functioning within a state of relaxed vigilance are more likely to produce affiliative laughter. Both processes may occur simultaneously. Consequently, laughter is best interpreted as a potentially informative indicator of regulatory conditions rather than as evidence of a specific causal mechanism. Future observational and longitudinal studies will be needed to clarify the temporal and causal relationships among laughter, communication patterns, psychological safety, burnout, and team functioning within care environments.

## Conclusion

Sustaining vigilance over time is a central challenge for human systems that operate under conditions of uncertainty and responsibility. In healthcare and other caregiving institutions, attention to potential risk must remain active while communication, trust, and coordination are preserved. When vigilance becomes chronic and unmodulated, however, it may shift toward rigidity, fear saturation, or defensive withdrawal, conditions that can undermine collective learning and relational coherence.

This Perspective has proposed the concept of relaxed vigilance as a regulatory condition in which vigilance remains active while preserving the relational and communicative capacities required for coordinated care. Within this framework, laughter is interpreted not simply as amusement but as a potential interactional signal that tension has not escalated into interpersonal threat. The framework does not claim that laughter causes relaxed vigilance; rather, it proposes that laughter may indicate or participate in regulatory conditions that allow attention to risk and relational coordination to coexist. Evidence from evolutionary, physiological, sociological, and healthcare teamwork research supports the plausibility of this interpretation while also underscoring the need for future empirical study.

Viewed sociologically, the presence or absence of humor can therefore be read as social data reflecting the regulatory conditions of human systems. When relational trust, communication openness, and coordinated attention remain intact, systems may be better able to sustain vigilance without collapse or fragmentation. Relaxed vigilance can therefore be understood as a regulatory condition that allows human systems to remain attentive to uncertainty while preserving the relational and communicative processes through which adaptive coordination becomes possible. In caregiving systems, the capacity to maintain relaxed vigilance may represent one way in which care systems uphold their duty of care while continuing to adapt under conditions of prolonged strain. More broadly, the concept of relaxed vigilance suggests that human systems depend not only on detecting threats but also on the capacity to regulate vigilance itself, preserving the relational and communicative conditions that allow collective action to continue under uncertainty.

## Data Availability

The original contributions presented in the study are included in the article/supplementary material, further inquiries can be directed to the corresponding author.
